# Efficient Nucleophilic Degradation of an Organophosphorus Pesticide “Diazinon” Mediated by Green Solvents and Microwave Heating

**DOI:** 10.3389/fchem.2018.00669

**Published:** 2019-01-14

**Authors:** Daniela Millán, Ricardo A. Tapia, Paulina Pavez

**Affiliations:** ^1^Facultad de Química, Pontificia Universidad Católica de Chile, Santiago, Chile; ^2^Centro Integrativo de Biologia y Quimica Aplicada, Universidad Bernardo O'Higgins, Santiago, Chile

**Keywords:** greener solvents, ionic liquids, organophosphate pesticides, ^31^P NMR, microwaves, ultrasound

## Abstract

An efficient strategy for the degradation of organophosphate pesticide Diazinon was investigated. In this work, ionic liquids, bio-based solvents, and two conventional organic solvents were used as reaction media. Kinetics studies by means of half-life (t_1/2_,h) were followed by ^31^P NMR and the products analyzed by GC-MS, HPLC-MS and NMR techniques. These results have shown that t_1/2_ values in ionic liquids were the lowest and also they were able to activate two electrophilic centers in Diazinon, whilst degradation in bio-based solvents occurred slowly by only an aromatic pathway. In addition, a study to estimate the influence of green activation techniques was carried out by using Ultrasound irradiation and Microwave heating in combination with greener solvents and two conventional organic solvents. Under Microwave heating, faster degradation than under ultrasound irradiation was found. Finally, considering both families of solvent used here and their behavior under green activation techniques, we propose that the more efficient way for degradation of Diazinon with piperidine is by microwave heating using ionic liquids as solvents.

## Introduction

Organophosphorus pesticides (OPPs) represent 38% of total pesticides used globally, due to their high insecticidal activity and other biological activities (Casida and Quistad, 2004; Singh, 2009). Considering the known toxicity of OPPs to humans, their presence in the environment is of great concern since most of them as well as their degradation products have been found both in surface and groundwater (Matouq et al., 2008). Therefore, degradation of these compounds is an important issue overall when some of the treatments to degrade OPPs may not be very efficient or are harmful to the environment due to the formation of by-products that have mild or acute toxicity (Ortiz-Hernández et al., 2003; Gan et al., 2006). Among the methods that have been developed for their degradation, microbiological and chemical processes are commonly used. Biological degradation takes place in soils when soil microorganisms or enzymes consume or break down pesticides (Richins et al., 1997; Deng et al., 2015), while chemical degradation occurs through reactions such as photolysis, hydrolysis, oxidation or nucleophilic attack by using O and N nucleophiles (Menger and Rourk, 1999; Kodaka et al., 2003; Bavcon Kralj et al., 2007). The latter is a promising alternative to other remediation approaches used for their destruction (Onyido et al., 2005; Rougier et al., 2010; Singh et al., 2015).

In this context, the solvent is essential to carry out this process, and the replacement of conventional organic solvents (COS) by a suitable alternative, has become one of the main topics of modern chemistry (Sheldon, 2005). In this respect, ionic liquids and bio-based sustainable solvents have lately appeared as the most promising approaches for current solvent innovation (Hallett and Welton, 2011; Yang et al., 2012; MacMillan et al., 2013). Both kinds of solvents have been used previously by our group to study nucleophilic substitutions reactions of Paraoxon and Fenitrothion and the results provided useful information for the appropriate degradation of organophosphate pesticides (Pavez et al., 2013, 2016).

On the other hand, not only the solvent plays a key role in organic reactions but time and energy efficiency are also important, overall when the requirement for a sustainable and safe process is gaining much attention. In this context, the use of irradiation methods can be a further way to meet the demands of the Green Chemistry principles. In this sense, Ultrasound (US) and Microwave (MW) technologies have been recently used as green activation techniques (Cravotto and Cintas, 2007) to improve the outcome of several organic reactions as well as an analytical technique to determine organic pollutants (Cravotto and Cintas, 2006; Papadopoulos et al., 2016). Ultrasound technique has been used to investigate the degradation of some OPPs, for instance, Matouq et al. studied the effect of high ultrasound irradiation frequency techniques in degradation of Diazinon in aqueous solution, concluding that the kinetics of degradation fit well with a pseudo-first-order process (Matouq et al., 2008). Zhang et al. treated different samples of apple juice which contained malathion and chlorpyrifos with ultrasonic irradiation, and their results showed that ultrasonic treatment was effective for the degradation of malathion and chlorpyrifos in apple juice (Zhang et al., 2010). In another study by Yao et al., investigated the mechanism of sonolithic degradation of Parathion, and they demonstrated that the degradation rate increased proportionally with an increase in ultrasonic intensity (Yao et al., 2010).

Additionally, the synergetic effect of ionic liquids in combination with US has been of great interest, due that they have demonstrated to generate improvements in yield, rate, and selectivity compared to classical chemistry, or products expected (D'Anna et al., 2012; Chatel and MacFarlane, 2014). Nevertheless, to the best of our knowledge, there is no report about the degradation of OPPs using ILs and greener bio-based solvents in combination with US or MW.

**Figure 1:**
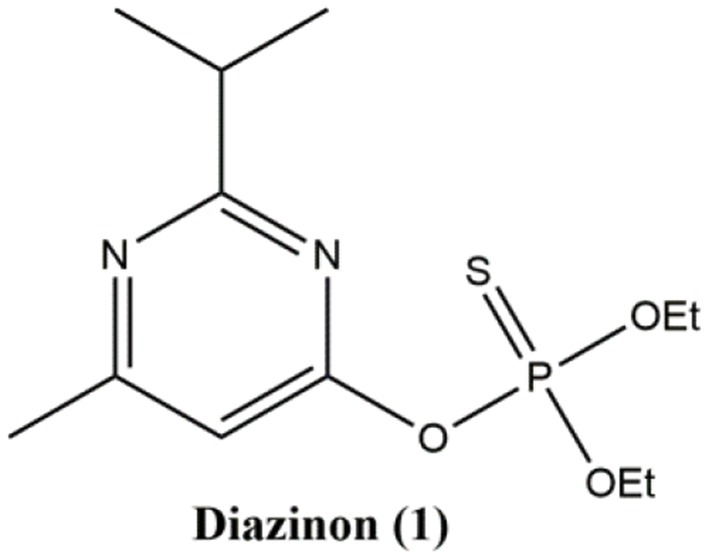
Pesticide under study.

**Scheme 1:**
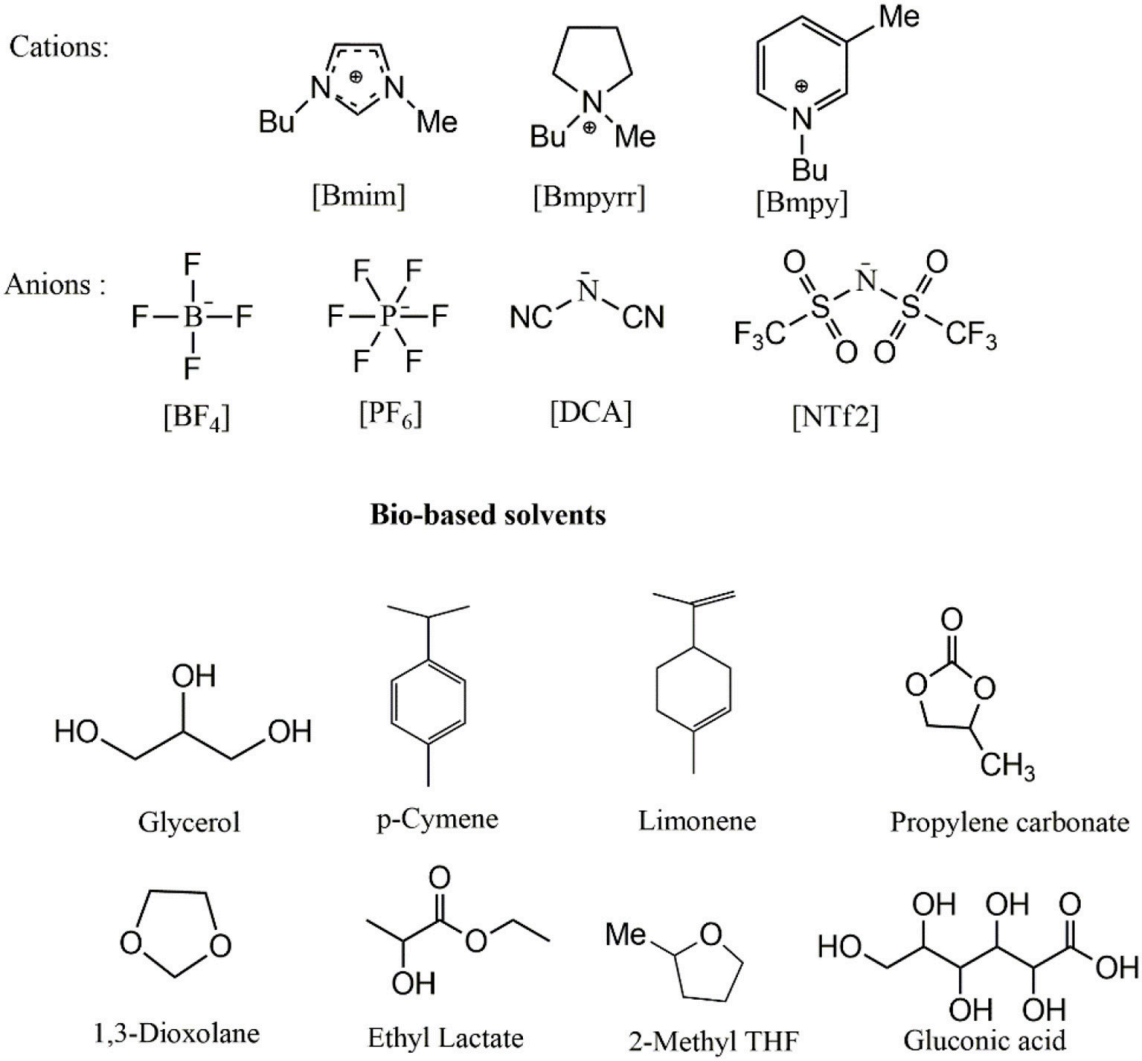
Greener solvents used in this study.

The purpose of this work was to study the influence of the solvent in the nucleophilic substitution reactions of organophosphate pesticide Diazinon **1** (Figure 1) with piperidine as a nucleophile, in six ILs and eighth bio-based solvents (Scheme 1). Additionally, we are motivated to compare the results obtained in ILs and bio-based solvents with those obtained under ultrasound and microwaves irradiation to search for a more efficient approach for the degradation of **1**.

## Experimental Section

### Materials

All bio-based solvents, conventional organic solvents (COS), piperidine and Diazinon® were purchased. All ionic liquids were dried before use on a vacuum oven at 70°C for at least 12 h and stored in a dryer under nitrogen and over calcium chloride. The water content in ILs was <0.1% by Karl Fisher titration.

### Kinetic Measurements

The kinetic study of the reaction of **1** with piperidine in all solvents was performed by ^31^P NMR obtained on a 400 spectrometer, following the disappearing of the signal of **1**, at least a 10-fold excess of total amine over the substrate was employed and each measurement was made in triplicate.

Typical ^31^P NMR kinetic experiment: an NMR tube containing 500 μL of solvent (COS, ILs or bio-based solvents) was thermostated at 25°C for 10 min. Then 10 μL of pesticide (0.5M in MeCN) and 40 μL of neat piperidine were added. Capillaries of deuterated water were used as reference solvent. The spectra were recorded at different reaction times and pseudo-first-order rate coefficients (*k*_obsd_) were found for the different reaction routes. The overall *k*_obsd_ values obtained for degradation of **1** were obtained by integration of the NMR signals and then plotted log (integration) vs. time. To obtain the rate constants for the product formation, we multiplied *k*_obsd_ by the fraction of each product relative to the total products. The molar concentrations of the products at the end of the reactions were obtained by quantitative analysis of each product.

### Microwave-Assisted Degradation

Microwave-assisted reactions were carried out in an Anton Paar Monowave 300 Microwave Synthesis Reactor (Anton Paar GmbH, Graz, Austria) in 10 mL sealed vials. To run a microwave experiment the same quantities of solvent, pesticide, and piperidine as kinetic measurements were used. Measurements were carried out at 50°C and 500 rpm, each measurement was made in triplicate. To evaluate the % of degradation of the pesticide the reaction mixture was analyzed by ^31^P NMR.

### Ultrasound-Assisted Degradation

The reactions were carried out in a thermostated ultrasonic cleaning bath (SB−3200 DTD) operating at a frequency of 40 kHz. The tank dimensions were 300 × 155 × 150 mm, with a liquid holding capacity of 6 L. The ultrasonic cleaner had an output power of 0–180 W through digital adjustment. The reactions were carried out in a round-bottomed flask of 20 mL capacity suspended at the center of the cleaning bath, 5 cm below the surface of the liquid. In a typical experiment 1,000 μL of solvent (COS, ILs or bio-based solvents), 20 μL of pesticide (0.5M in MeCN) and 80 μL of neat piperidine were added to the round-bottomed flask and then irradiated by 1 h. To evaluate the % of degradation of the pesticide the reaction mixture was analyzed by ^31^P NMR. Each measurement was made in triplicate.

### Electrospray Ionization Mass Spectrometry (ESI-MS)

**Scheme 2:**
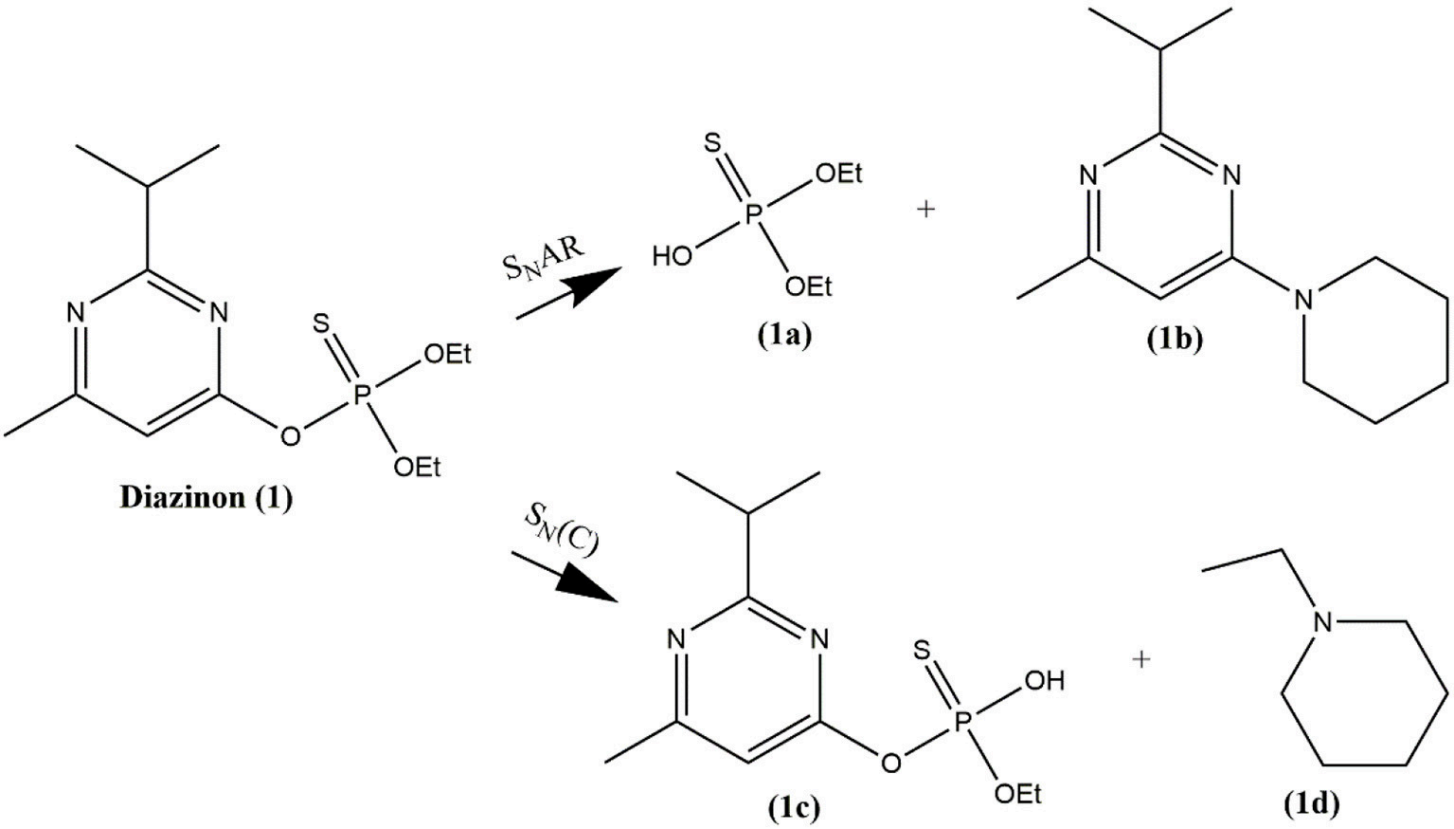
Reaction pathways for degradation of **1** by piperidine in all ionic liquids, DMSO and MeCN.

The detection of Compounds **1a**, **1b**, **1c**, and **1d** for degradation of **1** (See Scheme 2), were identified by an ABSciex Triple Quad 4500 (UHPLC-MS/MS) mass spectrometer equipped with a Turbo Ion Spray (AB Sciex) ion source. A microsyringe pump delivered the mixed reaction of **1** with piperidine in DMSO at infinite time dissolved in 10% (vol/vol) acetonitrile into the ESI source at a flow rate of 10 μL/min. ESI and the QQ (linear trap) mass spectrometer were operated in the negative-ion mode for detecting **1a** and **1c** and the positive mode for **1b** and **1d** by using the multiple reaction monitoring (MRM) scan types. Main conditions: curtain gas nitrogen flow = 10 mL min-1; ion spray voltage = −4,500 eV; declustering potential = −60 eV; entrance potential = −10 eV; collision cell exit potential = −12 eV; source temperature was set at 300°C and source gas GS1 and GS2 were set to 12 and 0, respectively. All data were acquired using Analyst 1.6.2 (AB Sciex).

## Results and Discussion

**Figure 2:**
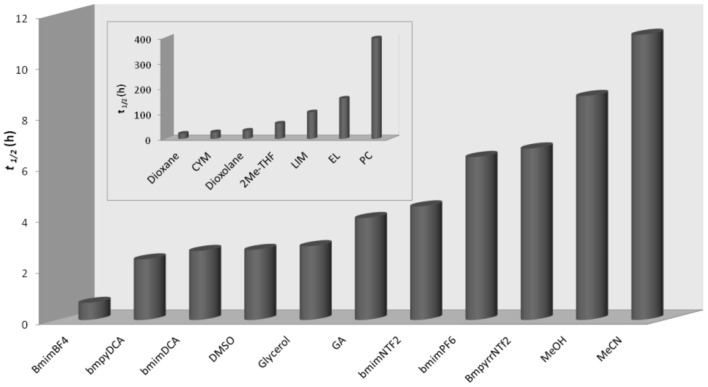
Half-life (t_1/2_,h) for degradation of **1** with piperidine (0.7M) in several solvents. Each measurement was made in triplicate.

To investigate the degradation of Diazinon in solvents labeled as “greener solvents,” we performed a kinetic study where by means of first-order rate constants (*k*_obsd_) obtained from nucleophilic attack of piperidine to **1** in Ionic liquids, bio-based solvents, and some conventional solvents; we calculated half-life (t_1/2_). The *k*_obsd_ were obtained by ^31^P NMR technique in the presence of total piperidine excess and calculated from the slope of a logarithmic plot of the ^31^P NMR area due to degradation of substrate **1** at different times [Table S1 in Electronic Supplementary Information (ESI)]. Figure 2 shows the t_1/2_ values obtained for degradation of **1** in all solvents used in this study.

As seen in Figure 2, the lowest t_1/2_ value was found in BmimBF_4_ (0.68 h), while the highest was in propylene carbonate (PC) (229 h). In fact, the ratio between highest and lowest is *ca* 327. In general, the highest values of t_1/2_ were found in bio-based solvents (inset of Figure 2), intermediate values in conventional solvents (MeCN and MeOH) and smallest in ionic liquids, DMSO, glycerol (GLY), and gluconic acid (GA). These results in ILs agree with a recent study where the degradation of Fenitrothion was investigated in the same family of solvents (Pavez et al., 2016). In that study, it was also found that ionic liquids were the best solvents to degradation Fenitrothion.

In order to analyze the solvent effects on the degradation of Diazinon, it is necessary to identify the electrophilic centers where the nucleophilic attack by piperidine is occurring.

**Figure 3:**
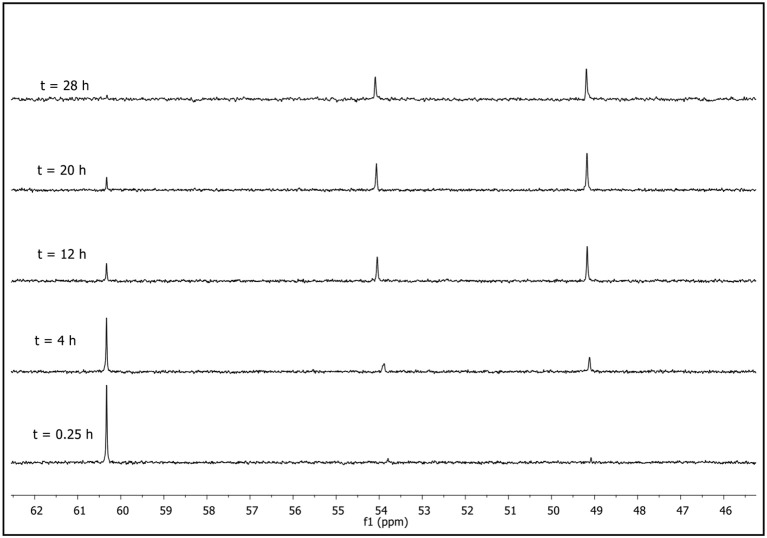
Progressive ^31^P NMR spectra obtained for degradation of **1** with piperidine (0.7M) at 25°C in BmpyrrNTF_2_.

Therefore, to have an insight into the mechanism of degradation of **1** we performed a product analysis of this reaction by NMR and GC-MS techniques (Figures S1–S3 in ESI). As an example, Figure 3 shows the ^31^P NMR spectra obtained for degradation of **1** with piperidine in BmpyrrNTF_2_ at different times.

As can be observed from Figure 3, the signal for pesticide **1** at 60.3 ppm decreases and two other phosphorylated signals increase simultaneously. These two new signals were assigned to the formation of the two phosphorylated species, diethyl thiophosphate (**1a**) and O-ethyl O-[4-methyl-6-(propan-2-yl)pyrimidin-2-yl]thiophosphate diester (**1c**) at approximately 54 and 48 ppm, respectively (shown in Scheme 2). Compound **1a** is formed by nucleophilic attack of piperidine at C-1 carbon of the aromatic ring of **1** via a S_N_Ar pathway, and **1c** by an attack at the aliphatic carbon of the O-ethyl group, through a bimolecular nucleophilic substitution (S_N_2(C)) mechanism. This behavior was found in all ionic liquids, DMSO and MeCN (see Figures S4–S10 in ESI), and the pathways are shown in Scheme 2.

In addition, the progressive ^31^P NMR spectra obtained for degradation of **1** with piperidine in CYM, LYM, EL, PC, MeOH, GLY, GLU (see Figures S11–S14, in ESI), showed only one phosphorylated signal that appears at approximately 58 ppm, which it was assigned to the formation of the phosphorylated species **1a** (Scheme 2). It is worth to mention that all ILs used in this study were capable of activating two electrophilic centers, aromatic and aliphatic carbons (S_N_Ar and S_N_2 routes), in contrast with bio-based solvents where degradation only takes place through S_N_2(C) route.

**Figure 4:**
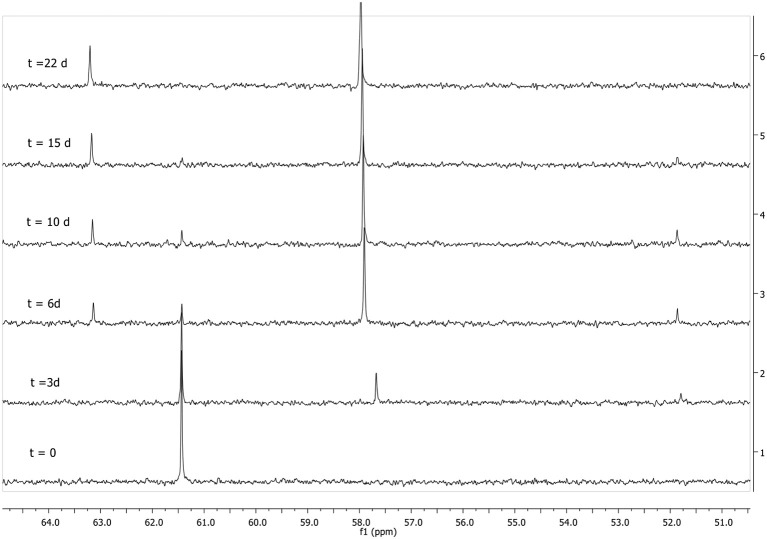
Progressive ^31^P NMR spectra obtained for degradation of **1** with piperidine (0.74M) at 25°C in 1,3-Dioxolane.

On the other hand, when 1,3-dioxolane, dioxane, and 2-MeTHF were the solvents as reaction media, the product distribution was different to those described in Scheme 2. As an example, Figure 4 shows the progressive ^31^P NMR spectra obtained for degradation of **1** in 1,3-dioxolane. It can be observed that while the signal of pesticide decreases (61 ppm) the same two signals assigned to the nucleophilic attack of piperidine to the aromatic carbon (57.5 ppm) and to the aliphatic carbon of **1** (51.5 ppm) increased. But, at very long reaction time it is possible to see a new signal at approximately 62.5 ppm, which increases at expenses of the signal at 51.5 ppm (Figure 4).

**Scheme 3:**
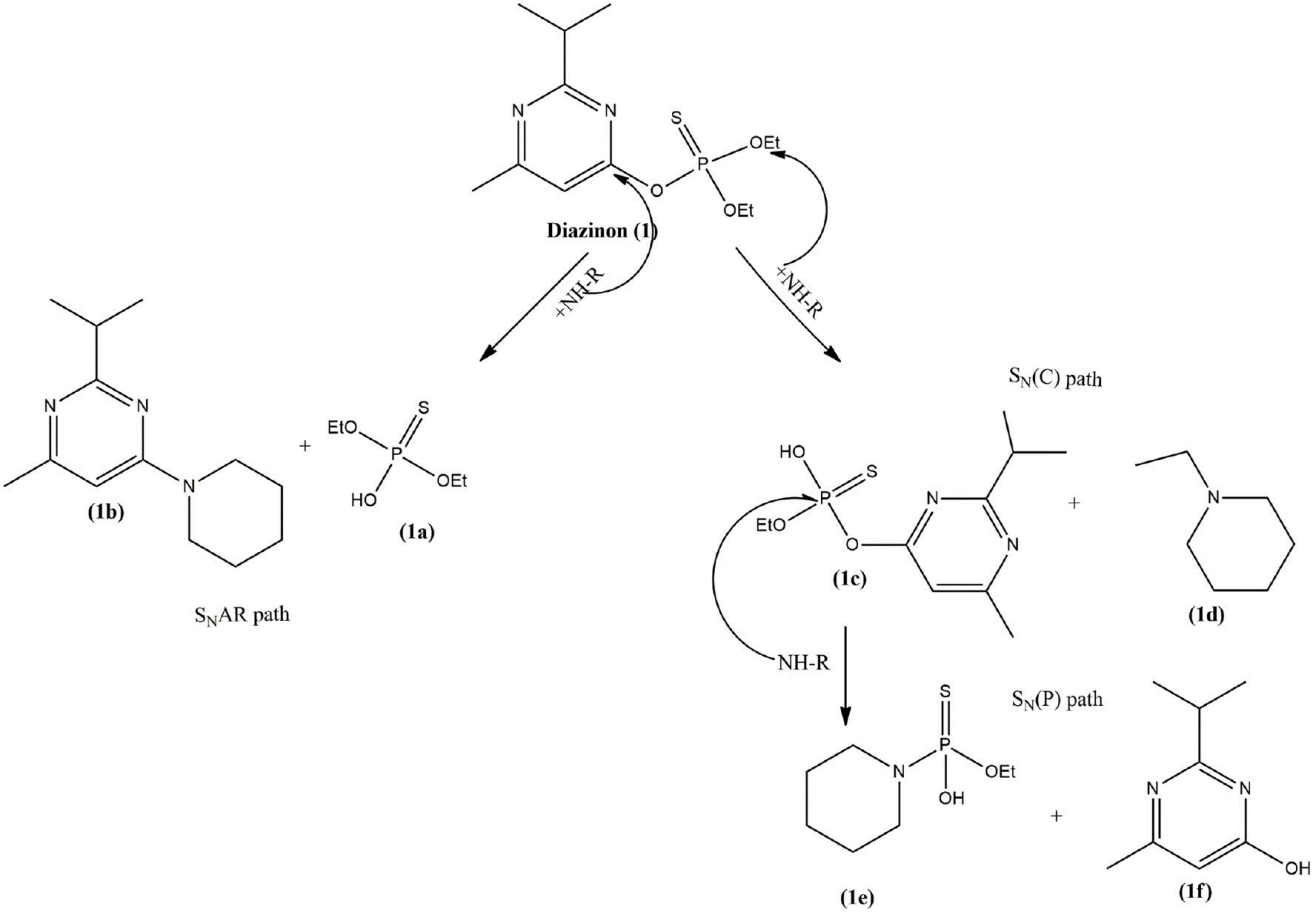
Reaction pathway for degradation of **1** by piperidine in 1,3-Dioxolane, Dioxane and 2-MeTHF.

This new phosphorylated species (**1e**) was attributed to the product formed by a new piperidine attack at the phosphorus atom of compound **1c**, as shown in Scheme 3. Progressive ^31^P NMR spectra obtained for degradation of **1** in Dioxane and 2-MeTHF are shown in Figures S15, S16 in ESI.

**Figure 5:**
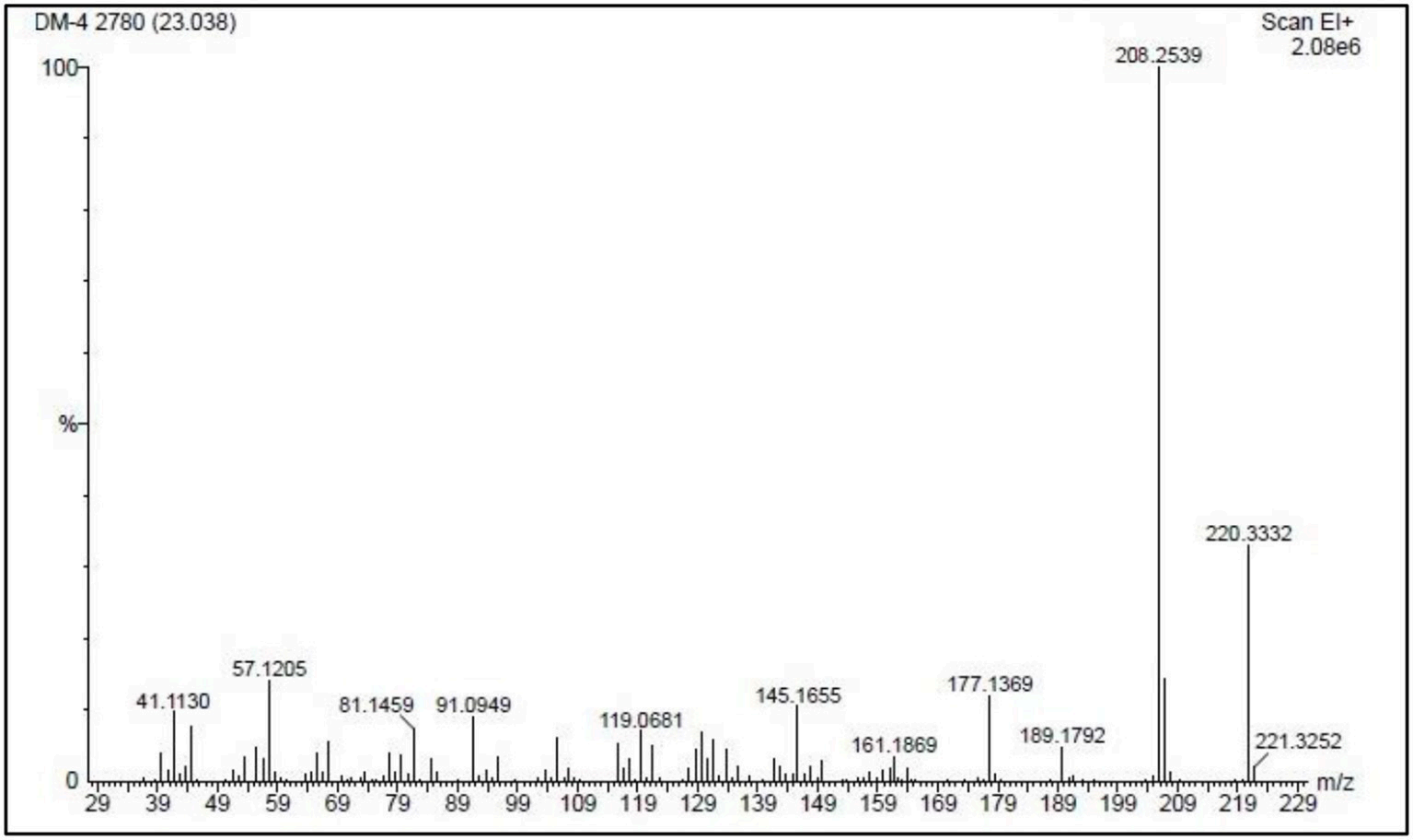
Mass spectrum of compound **1e** (r.t. = 23.04min, m/z = 208.25) from an extract of the reaction of **1** with piperidine in 2-MeTHF at 25°C.

The presence of compound **1e** as a reaction product was confirmed by GC/MS by means of an extraction with diethyl ether from the reaction medium and this extract was analyzed by GC/MS (compound **1e:** r.t. = 23.04 min, m/z = 208.25), the principal fragments are shown in Figure 5.

**Figure 6:**
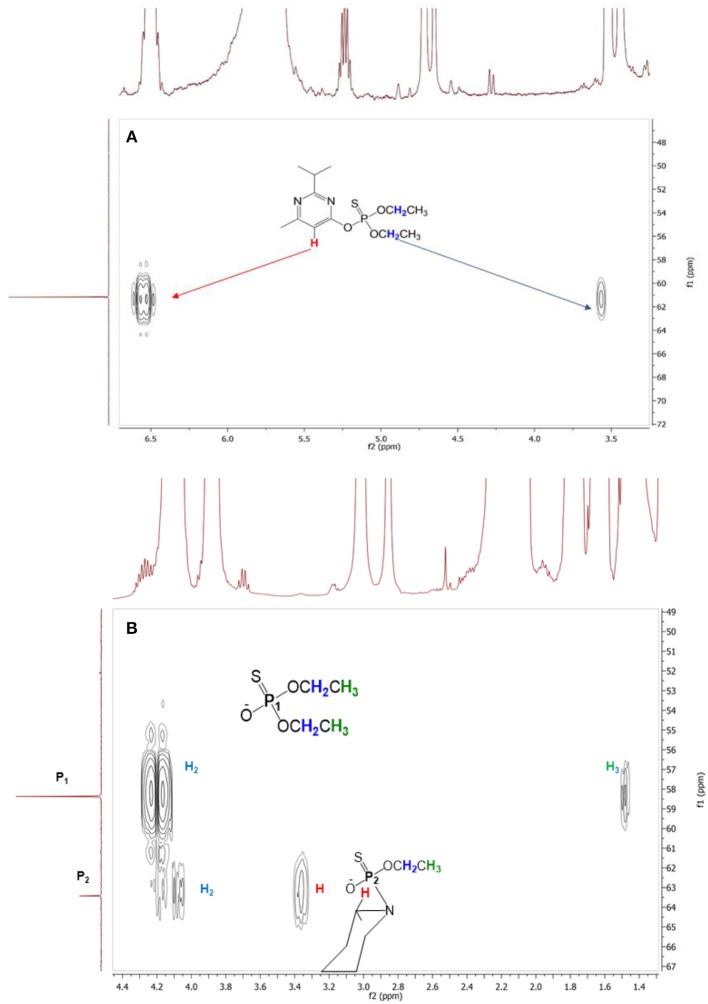
2D ^31^P-^1^H HMBC experiment of the reaction of Diazinon with piperidine in 2-Me THF at 25°C at the beginning **(A)** and at the end **(B)** of the reaction.

In addition, in order to confirm the structure of compound **1e** we also performed a 2D ^31^P-^1^H HMBC experiment. Figure 6 shows the HMBC spectra obtained at the beginning (A) and at the end of the reaction (B) of Diazinon with piperidine in 2Me-THF as a solvent. As we can see, the only aromatic proton of Diazinon is lost when reacting with piperidine. When 2Me-THF is the solvent two phosphoryl compounds are observed at the end of the reaction: (a) compound **1a** where the correlation with aliphatic protons of Ethyl moiety are clear and (b) compound **1e** where the loss of one Ethyl group is evidenced at around 1.4 ppm, and the new correlation of a phosphorous atom with a proton is found at ~3.4 ppm which is assigned at one of the protons of piperidine.

**Figure 7:**
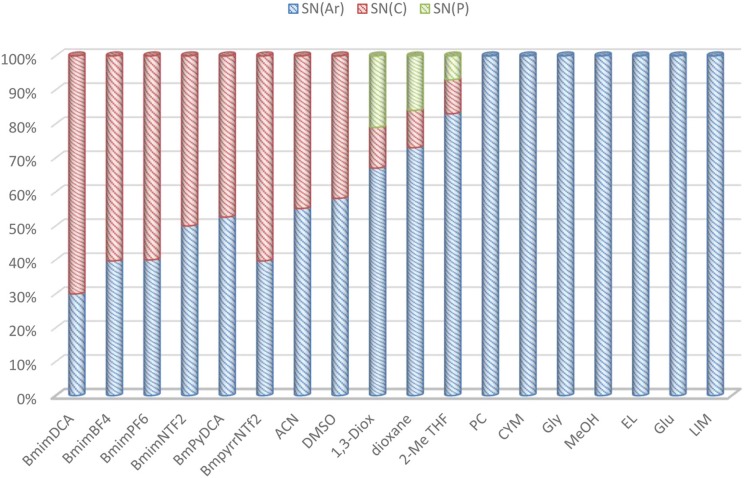
Relative nucleophilic attack of piperidine to **1** in ILs, bio-based and conventional solvents. Attack of piperidine at phosphorus atom of **1c** (see scheme 2) by S_N_2(P) pathway (green columns), C-1 aromatic carbon by S_N_Ar pathway (blue columns), and aliphatic carbon by S_N_2(C) pathway (red columns).

On the other hand, from the integration of the ^31^P NMR signals of the products formed by piperidine attack to the different electrophilic centers of **1** in all solvents used in this study (see Figure 4 and Figures S4–S16 in ESI), the relative products distribution was calculated in the different reaction media. Figure 7 shows this behavior schematically.

It is clear from Figure 7 that there are three different behaviors, and the selectivity of this reaction is strongly dependent on the nature of the solvent. ILs promote both S_N_Ar and S_N_2(C) pathways (blue and red columns, respectively), and the highest selectivity for S_N_2(C) route was observed with BmimDCA (70%). For those ILs sharing Bmim cation preference for S_N_2(C) path decreases on increasing the size of the anion from DCA>BF_4_>PF_6_>NTf_2_. In addition, fixing anion DCA and changing from imidazolium (Bmim) to pyridinium cation (Bmpy), selectivity to S_N_2(C) route decreases. The enhanced selectivity to S_N_Ar could be explained by means of higher aromaticity of Bmpy cation compared to aromaticity of Bmim cation. These results would suggest a higher π stacking interaction between aromatic cation of the IL and the leaving group of the pesticide, favoring the S_N_Ar route. Note that the reaction in 1,3-dioxolane, dioxane and 2-MeTHF solvents have a different behavior, and in addition to S_N_Ar and S_N_2(C) pathways, a nucleophilic attack of piperidine to the phosphorus atom of **1c** by S_N_2(P) (green columns) was observed. This three solvents are cyclic ethers present some structural similarities and show similar behaviors in S_N_Ar reactions (Mancini et al., 1984).

In order to account the influence of the solvent in organic reactions Kamlet–Taft parameters (α, β, and π^*^) are frequently used, which assess hydrogen bond donating ability (β), hydrogen bond accepting ability (α), and a combination of dipolarity and polarizability (π^*^) (Kamlet et al., 1977, 1983).

Thus, to rationalize the influence of the solvent on the t_1/2_ of degradation of Diazinon, we have performed a multiparameter linear solvation energy relationship (LSER) between the logarithm of t_1/2_ values and several empirical solvent parameters. Considering the reaction mechanism shown in Scheme 2, we have obtained t_1/2_ values for S_N_Ar and S_N_2(C) reaction routes by using the relative product distribution of Figure 7 and Table S2. The t_1/2_ values calculated for the different reaction pathways and the solvent parameters for each solvent used are reported in Tables S1, S3 in ESI, respectively. Table 1 shows the multiparametric regression analyses including Kamlet-Taft solvent parameters described above, viscosity (η), Hildebrandt's parameter (δ_H_), and $E_{N}^{T}$ polarity parameter. Each parameter is empirically obtained and has been measured in a wide range of solvents, including ionic liquids and bio-based solvents (Jessop et al., 2012).

**Table 1:** Statistical data from the multiparametric regression procedure including viscosity (η), α, β, π^*^ Kamlet–Taft, Hildebrand solubility ($\delta_{\text{H}}^{2}$) and $E_{N}^{T}$ polarity parameters for solvents used in degradation of **1** by S_N_Ar route.

**Equation**	**^a^F**	**^b^R^**2**^**	**α**	**β**	π^*^	**$\delta_{\text{H}}^{2}$**	**η**	**$E_{N}^{T}$**
1	0.49	−0.23	−15.5(47.4)	−53.7(55.1)	17.7(59.2)			
2	56.1	0.94	−5.9(16.1)	−9.61(20.7)	−195(18.8)	9·10^−3^(0.014)		
3	2.64	0.29			−138(69.3)	0.21(0.09)		
4	2.97	0.42			−238(89.5)	0.24(0.08)		113.6(73.1)
5	58.9	0.91			−175(24.1)			−15.0(25.1)
6	35.7	0.91			−174(25.5)		4.96·10^−3^(0.017)	−17.8(28.3)
7	302	0.95			−194.3(11.2)			

a *Statistical F*.

b Correlation coefficient.

*Standard deviations are given in parentheses*.

**Figure 8:**
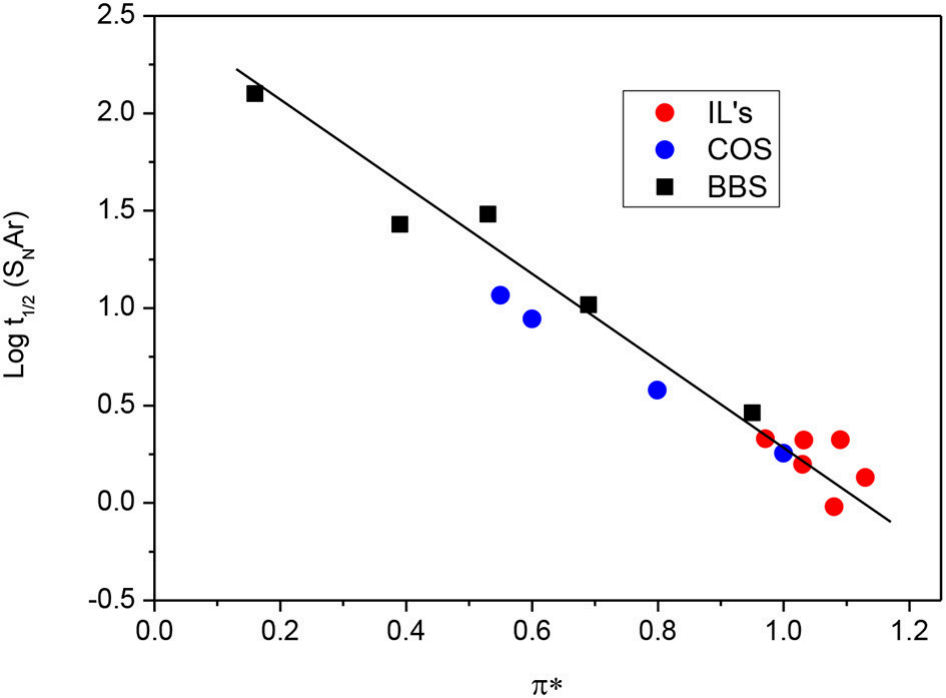
plot of log t_1/2_ against π* parameter, obtained for the degradation of **1** by S_N_Ar route in several solvents.

As we can see in Table 1 several equations were tested varying the number of parameters for the S_N_Ar route. The best correlation was found for a S_N_Ar pathway between log t_1/2_ values and π^*^ parameter, Figure 8 shows this comparison. On the other hand Table S3 in ESI shows the same procedure for the S_N_2(C) pathway.

As we can see from Figure 8, degradation of pesticide Diazinon occurs faster via S_N_Ar in solvents with high π^*^ values. This could be explained because S_N_Ar route involves formation of Meisenheimer complex during addition-elimination process which leads to a dipolar intermediate which is large and highly polarizable, in addition, π^*^ parameter is related to the ability of a bulk solvent to stabilize a charged or a dipolar solute by means of charge-dipole or dipole-dipole interactions (Martinez et al., 1986), therefore, seems correct a good correlation between π^*^ parameter and log t_1/2_ for the aromatic route. The same trend it has been described for rate and equilibrium constant for the formation of Meisenheimer complexes when changing from polar protic solvents to dipolar aprotic solvents (Vinson et al., 1983; El Guesmi et al., 2013; Gazitúa et al., 2014).

Finally, with the aim of achieving a more degradation, we have performed a study to investigate the effect of green activation techniques on the degradation of this pesticide in some conventional and greener solvents. Thus, the reaction under study was carried out in three ionic liquids, four bio-based solvents, and two conventional solvents under microwave heating and ultrasonic waves. We tried to perform the reactions under MW and US during the same period of time, but after 1 h of MW irradiation many of the reactions had finished, for that reason, we chose to perform the experiments for 30 min at 50°C. To contrast with activation techniques, the reactions were carried out in silent conditions, *ie*, no irradiation at room temperature to 60 min.

**Figure 9:**
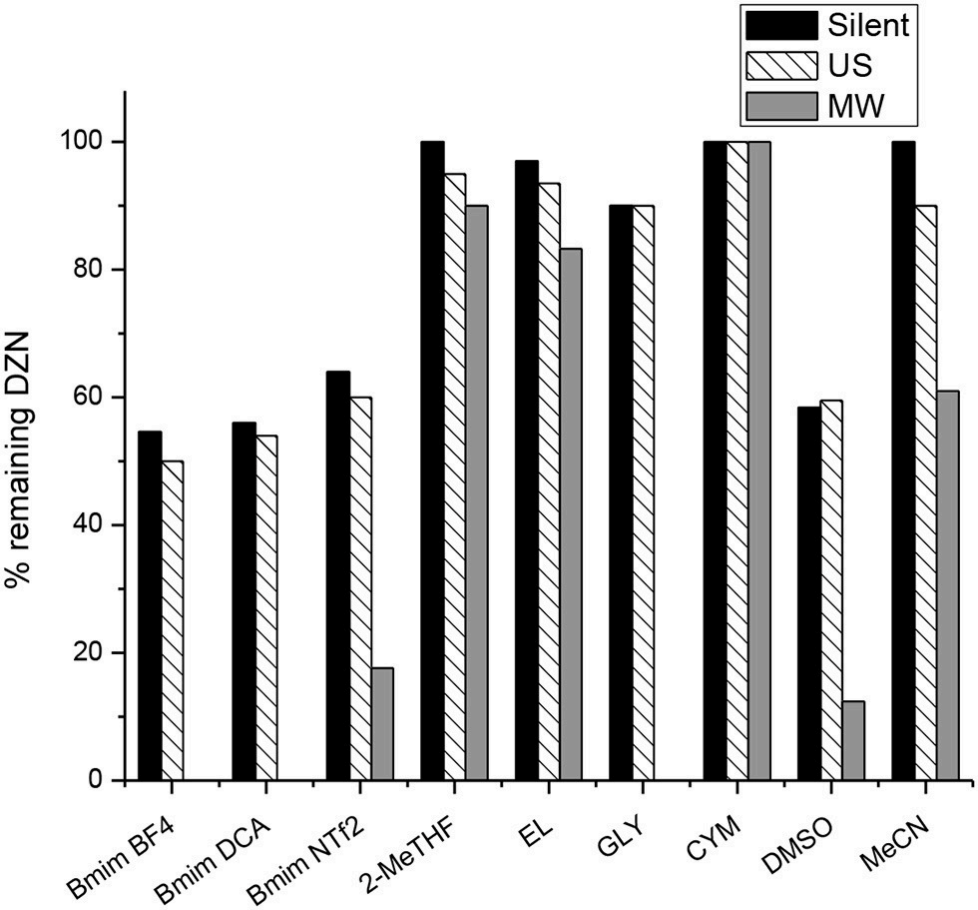
Percentage (%) of resting Diazinon after 30min of microwave (MW) heating and 60min of ultrasound (US)waves and silent conditions.

Figure 9, illustrates the percentage (%) of resting Diazinon calculated by integration of signals of ^31^P NMR after 60 min of silent conditions, 60 min of ultrasound waves and 30 min of microwave heating at 50°C.

The comparative graph (Figure 9) shows that the best technique to degrade Diazinon was by MW irradiation, whilst the best solvents to carry out the degradation under microwave heating were Ionic Liquids and Glycerol. It can be observed that similar % of resting Diazinon are obtained in silent conditions and with the ultrasound bath. These results can be attributed to the low ultrasonic power delivered by the bath in comparison with MW.

Degradation assisted by US and MW in bio-based solvents, with exception of glycerol, shown that it occurs very slowly independent of the activation method used. For example, when CYM is the solvent there is no degradation during the experiment time, even with MW irradiation. It is worth to highlight the contrasting behavior of glycerol under US and MW irradiation in this reaction. Since glycerol is a polar molecule with high viscosity, high acoustic impedance and a loss tangent of 0.651 in addition to its surface tension, it was expected a comparable performance under both activation techniques (Cintas et al., 2014; García et al., 2014). However, US waves seem not to affect degradation of Diazinon in this solvent. Despite the physical properties of glycerol enhances the cavitational collapse the viscosity is a drawback, which is usually overcome by conventional heating or microwave activation (Kappe, 2004).| In this context, a great performance is observed under MW heating where 100% of degradation is obtained after 30 min of heating.

On the other hand, when DMSO and MeCN are the reaction media, degradation under US and silent conditions are quite close in these solvents, but again the polarizable DMSO takes advantage when heating under MW.

Regarding the ionic liquids used under MW irradiation, BmimBF_4_ and BmimDCA showed a complete degradation in 30 min of heating whilst when BmimNTF_2_ is the solvent still remains 18% of Diazinon after the time of irradiation.

Finally, results obtained in ILs and GLY with MW are in accordance with those reports where a synergetic behavior of ILs and GLY in combination with MW have been found (Hu et al., 2010; Cintas et al., 2014). Due to MW heat up molecules or ions through friction, the presence of ions and/or polar molecules is necessary for substances to be heated in the microwave (Hoffmann et al., 2003).

## Conclusions

Degradation of Diazinon by nucleophilic reactions with piperidine in ILs, bio-based solvents and conventional solvents have been investigated at room temperature and under Ultrasound and Microwave irradiation. In silent conditions, the best solvents for degradation of Diazinon were ILs, which not only have the lowest t_1/2_ values but show selectivity to the aliphatic route, reaching 70% of preference in BmimDCA. The S_N_2(C) pathway leads to phosphate products less lipophilic and therefore less toxic to the human being.

A comparison between Ultrasound and Microwaves activation techniques shows that degradation of Diazinon proceed faster under Microwave irradiation and furthermore it was found a synergetic combination when using ionic liquids and glycerol as reaction media.

Finally, it is difficult to choose the best solvent to carry out the degradation since both ionic liquids and glycerol have a good performance under MW heating, but considering that ionic liquids are versatile solvents which can be tuned to specific reactions, we conclude that the more efficient way for the degradation of Diazinon is the microwave-assisted using ionic liquids as solvents.

## Author Contributions

DM contributed conception and design of the study as well as experimental work. PP and DM organized the database and performed the statistical analysis. DM wrote the first draft. All authors contributed to manuscript revision, read and approved the submitted version.

### Conflict of Interest Statement

The authors declare that the research was conducted in the absence of any commercial or financial relationships that could be construed as a potential conflict of interest. The handling Editor declared a past co-authorship with one of the authors DM.

## Supplementary Material

### Electronic Supplementary Information (ESI)

Stacked ^31^P NMR plot for the reaction of Diazinon with piperidine in all solvents, GC/MS and ESI-MS chromatograms and mass spectra, experimental procedure and kinetics results are available.
