# Supplementary material for: Efficient Nucleophilic Degradation of an Organophosphorus Pesticide “Diazinon” Mediated by Green Solvents and Microwave Heating
Source: Front Chem. 2019 Jan 14;6:669. doi: 10.3389/fchem.2018.00669 (PMC6340133; doi:10.3389/fchem.2018.00669)
Supplement: Supplementary file 1 [file Table_1.docx]

Sustainable degradation of organophosphorate pesticide “Diazinon”

Daniela Millán*, Paulina Pavez and Ricardo Tapia

*^1^Facultad de Química. Pontificia Universidad Católica de Chile. Casilla 306, Santiago 6094411, Chile.*

*^2^Universidad Bernardo O’Higgins, CIBQA, General Gana 1702, Santiago, Chile*

**Author Information**

*Corresponding author e-mail: daniela.millan@ubo.cl

**Table of contents**

| **Table S1**. Viscosity, Kamlet–Taft descriptors, Hildebrand parameters and first-order rate constants (*k*_obsd_) for degradation of **1** with piperidine. |  |
| --- | --- |
| Figure S1: ESI-MS/MS(+) of the compound **1d** of *m/z* 113.1, from a reaction of **1** with piperidine in water |  |
| Figure S2: ESI-MS/MS(+) of the compound **1b** of *m/z* 218.1, from a reaction of **1** with piperidine in DMSO. |  |
| Figure S3: ESI-MS/MS(-) of the compounds **1a** and **1c,**  of *m/z* 169.1 and 276.07 respectively, from a reaction of **1** with piperidine in DMSO. |  |
| Figure S4: Stacked ^31^P-NMR plot for the reaction of **1** with piperidine in [Bmpy]DCA at 25°C. |  |
| Figure S5: Stacked ^31^P-NMR plot for the reaction of **1** with piperidine in [Bmim]BF_4_ at 25°C. |  |
| Figure S6: Stacked ^31^P-NMR plot for the reaction of **1** with piperidine in [Bmim]NTf_2_ at 25°C |  |
| Figure S7: Stacked ^31^P-NMR plot for the reaction of **1** with piperidine in [Bmim]DCA at 25°C. |  |
| Figure S8: Stacked ^31^P-NMR plot for the reaction of **1** with piperidine in [Bmim]PF_6_ at 25°C. |  |
| Figure S9: Stacked ^31^P-NMR plot for the reaction of **1** with piperidine in DMSO at 25°C. |  |
| Figure S10: Stacked ^31^P-NMR plot for the reaction of **1** with piperidine in ACN at 25°C |  |
| Figure S11: Stacked ^31^P-NMR plot for the reaction of **1** with piperidine in CYM at 25°C |  |
| Figure S12: Stacked ^31^P-NMR plot for the reaction of **1** with piperidine in LIM at 25°C. |  |
| Figure S13: Stacked ^31^P-NMR plot for the reaction of **1** with piperidine in EL at 25°C. |  |
| Figure S14: Stacked ^31^P-NMR plot for the reaction of **1** with piperidine in MeOH at 25°C. |  |
| Figure S15: Stacked ^31^P-NMR plot for the reaction of **1** with piperidine in 1,4-dioxane at 25°C. |  |
| Figure S16: Stacked ^31^P-NMR plot for the reaction of **1** with piperidine in 2-MeTHF at 25°C. |  |
| Table S2: Relative products distribution (%) for the nucleophilic attack of piperidine to **1** in the different solvent used. |  |
| Table S3: Percentage (%) of the remaining DZN after a hour of reaction under US and silent conditions, and 30 min under MW irradiation. (0.74M piperidine) |  |
| Table S4: : Percentage (%) of the remaining DZN after a hour of reaction under US and silent conditions, and 30 min under MW irradiation. (0.74M piperidine) |  |

**Table S1**. Viscosity, Kamlet–Taft descriptors, Hildebrand parameters and first-order rate constants (*k*_obsd_) for degradation of **1** with piperidine.

| Solvent | η, _25 ºC_ (mPa s) | $E_{T}^{N}$ | α | β | π* | δ_H_ | *k*_obs_/10^6^ (s^-1^) |
| --- | --- | --- | --- | --- | --- | --- | --- |
| MeCN | 0.34 | 0.47 | 0.35 | 0.37 | 0.80 | 24.2 | 17.2 |
| Dioxane | 1.18 | 0.16 | 0.00 | 0.37 | 0.55 | 20.5 | 9.17 |
| MeOH | 0.55 | 0.76 | 0.98 | 0.66 | 0.60 | 29.7 | 21.9 |
| DMSO | 1.96 |  | 0.00 | 0.74 | 1.00 | 26.6 | 70.0 |
| CYM | 0.82 |  | 0.00 | 0.13 | 0.39 | 17.4 | 7.17 |
| LYM | 0.85 |  | 0.00 | 0.00 | 0.16 | 15.1 | 1.53 |
| 2-MeTHF | 0.46 | 0.18 | 0.00 | 0.58 | 0.53 | 16.9 | 3.15 |
| EL | 2.53 | 0.63 | 0.69 | 0.52 | 0.82 | 20.2 | 1.21 |
| PC | 2.50 | 0.47 | 0.00 | 0.38 | 0.90 | 27.2 | 0.84 |
| GLU | 18 |  | - | - | - | - | 48.1 |
| DIOX | 0.56 | 0.38 | 0.00 | 0.45 | 0.69 | 20.9 | 5.83 |
| GLY | 945 | 0.81 | 0.88 | 0.66 | 0.95 | 36.2 | 66.5 |
| [Bmim]BF_4_ | 219 | 0.66 | 0.61 | 0.38 | 1.08 | 24.3 | 282 |
| [Bmim]DCA | 29.9 | 0.64 | 0.47 | 0.71 | 1.13 | 27.9 | 71.1 |
| [Bmim]PF_6_ | 450 | 0.67 | 0.63 | 0.21 | 1.03 | 25.8 | 30.1 |
| [Bmim]NTf_2_ | 69 | 0.55 | 0.94 | 0.23 | 1.09 | 26.7 | 43.2 |
| [Bmpyrr]NTf_2_ | 85 | 0.54 | 0.41 | 0.24 | 0.97 | 21.2 | 28.6 |
| [Bmpy]DCA |  |  |  |  |  |  | 81.0 |


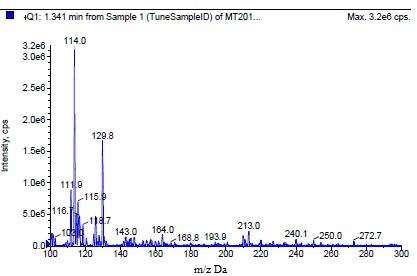


Figure S1: ESI-MS/MS(+) of the compound **1d** of *m/z* 113.1, from a reaction of **1**with piperidine in water.


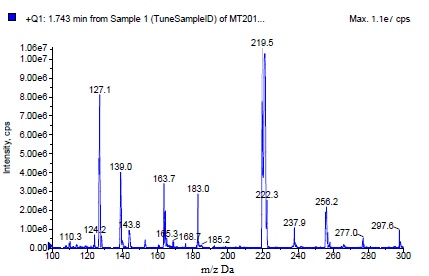


Figure S2: ESI-MS/MS(+) of the compound **1b** of *m/z* 218.1, from a reaction of **1**with piperidine in DMSO.


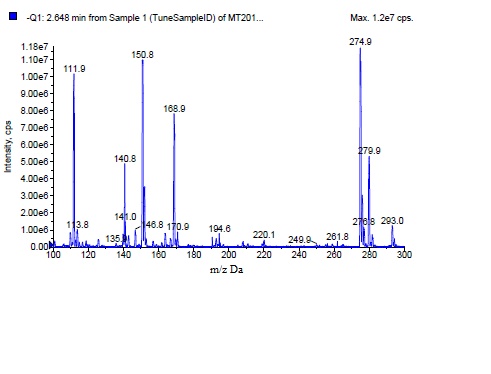


Figure S3: ESI-MS/MS(-) of the compounds **1a** and **1c,**  of *m/z* 169.1 and 276.07 respectively, from a reaction of **1** with piperidine in DMSO.

Figure S4: Stacked ^31^P-NMR plot for the reaction of **1** with piperidine in [Bmpy]DCA at 25°C.

Figure S5: Stacked ^31^P-NMR plot for the reaction of **1** with piperidine in [Bmim]BF_4_ at 25°C.

Figure S6: Stacked ^31^P-NMR plot for the reaction of **1** with piperidine in [Bmim]NTf_2_ at 25°C.

Figure S7: Stacked ^31^P-NMR plot for the reaction of **1** with piperidine in [Bmim]DCA at 25°C.

Figure S8: Stacked ^31^P-NMR plot for the reaction of **1** with piperidine in [Bmim]PF_6_ at 25°C.

Figure S9: Stacked ^31^P-NMR plot for the reaction of **1** with piperidine in DMSO at 25°C.

Figure S10: Stacked ^31^P-NMR plot for the reaction of **1** with piperidine in ACN at 25°C.


Figure S11: Stacked ^31^P-NMR plot for the reaction of **1** with piperidine in CYM at 25°C.

Figure S12: Stacked ^31^P-NMR plot for the reaction of **1** with piperidine in LIM at 25°C.

Figure S13: Stacked ^31^P-NMR plot for the reaction of **1** with piperidine in EL at 25°C.

Figure S14: Stacked ^31^P-NMR plot for the reaction of **1** with piperidine in MeOH at 25°C.

Figure S15: Stacked ^31^P-NMR plot for the reaction of **1** with piperidine in 1,4-dioxane at 25°C.

Figure S16: Stacked ^31^P-NMR plot for the reaction of **1** with piperidine in 2-MeTHF at 25°C.

Table S2: Relative products distribution (%) for the nucleophilic attack of piperidine to **1** in the different solvent used.

| Solvent | % S_N_Ar | %S_N_( C) | %S_N_(P) |
| --- | --- | --- | --- |
| bmimDCA | 30 | 70 | -- |
| bmimPF6 | 40 | 60 | -- |
| bmimNTF2 | 50 | 50 | -- |
| bmpyrrNTf2 | 39.7 | 60.3 | -- |
| bmpyDCA | 52.6 | 47.4 | -- |
| MeCN | 55.1 | 44.9 | -- |
| dioxane | 80.6 | -- | 19.4 |
| Cym | 100 | -- | -- |
| Lym | 100 | -- | -- |
| Me-THF | 83 | 10 | 7 |
| EL | 100 | -- | -- |
| Glu | 100 | -- | -- |
| DMSO | 58.1 | 41.9 | -- |
| MeOH | 100 | -- | -- |
| 1,3 diox | 67 | 12 | 21 |
| PC | 100 | -- | -- |
| BmimBF4 | 39.7 | 60.3 | -- |
| water | 100 | -- | -- |
| GLY | 100 | -- | -- |

Table S3: Statistical data from the multiparametric regression procedure including viscosity (η), α, β, π* Kamlet–Taft, Hildebrand solubility () and $E_{N}^{T}$ polarity parameters for solvents used in degradation of **1**.

| Without considering reaction pathways | | | | | | | | |  |
| --- | --- | --- | --- | --- | --- | --- | --- | --- | --- |
| Equation | **^a^F** | **^b^R^2^** | **α** | **β** | **π*** | **δ_H_^2^** | **η** | $\boldsymbol{E}_{\boldsymbol{N}}^{\boldsymbol{T}}$ | **intercept** |
| 8 | 0.9 | -0.05 | -1.12(1.12) | -0.46(1.60) | -1.79(1.54) | -0.006(0.077) | -6.02E^-4^(0.00107) | 1.77(2.78) | 2.64(0.35) |
| 9 | 3.1 | 0.3 | -0.42(0.47) | -0.17(0.77) | -1.27(0.68) |  |  |  | 2.4(0.40) |
| 10 | 2.2 | 0.24 | -0.3(0.57) | 0.009(0.92) | -1.15(0.77) | -0.02(0.05) |  |  | 2.65(0.34) |
| 11 | 5.7 | 0.38 |  |  | -1.2(0.71) | -0.032(0.037) |  |  | 2.86(0.68) |
| 12 | 2.6 | 0.21 |  |  | -1.4(1.11) |  |  | -0.58(1.17) | 2.76(0.78) |
| 13 | 42 | 0.75 |  |  | -1.79(0.27) |  |  |  |  |
| Only for S_N_(P) route | | | | | | | | | |
| 14 | 1.65 | 0.20 | 0.08(0.79) | -0.40(0.92) | -1.37(1.00) |  |  |  | 1.79(0.65) |
| 15 | 2.11 | 0.22 |  |  | -1.46(1.13) | 0.02(0.10) |  |  | 1.24(0.85) |
| 16 | 1.09 | 0.08 |  |  |  | 0.047(0.08) | 7E-4(0.012) | -2.65(2.14) | 0.60(1.17) |
| 17 | 5.81 | 0.35 |  |  | 1.19(0.49) |  |  |  | 1.48(0.47) |
| 18 | 1.21 | 0.01 | -0.012(0.85) | -0.75(1.04) | -2.08(1.49) | 0.053(0.07) |  |  | 1.34(0.95) |
|  |  |  |  |  |  |  |  |  |  |
|  |  |  |  |  |  |  |  |  |  |
| ^a^ Statistical F. ^b^ Correlation coefficient. ^c^ Standard deviations are given in parentheses | | | | | | | | |  |

Table S4: Percentage (%) of the remaining DZN after a hour of reaction under US and silent conditions, and 30 min under MW irradiation. (0.74M piperidine)

| Solvent | S (25°C) | US (25°C) | MW (50°C) |
| --- | --- | --- | --- |
| Bmim BF_4_ | 54.6 | 50 | 0 |
| Bmim DCA | 56 | 54 | 0 |
| Bmim NTf_2_ | 64 | 60 | 17.6 |
| 2-MeTHF | 100 | 95 | 90 |
| EL | 97 | 93.5 | 83.3 |
| GLY | 90 | 80 | 0 |
| CYM | 100 | 100 | 100 |
| DMSO | 58.4 | 59.5 | 12.4 |
| H_2_O | 0 | 0 | 0 |
| MeCN | 100 | 90 | 61 |
